# Environmental Contaminants, Iron Deficiency, and Iron-Deficiency Anemia: A Review of the Literature

**DOI:** 10.1155/sci5/5007983

**Published:** 2025-09-08

**Authors:** Rebecca Lichtler, Michael Cowley

**Affiliations:** ^1^Department of Biological Sciences, North Carolina State University, Raleigh, North Carolina, USA; ^2^Center for Human Health and the Environment, North Carolina State University, Raleigh, North Carolina, USA; ^3^Toxicology Program, North Carolina State University, Raleigh, North Carolina, USA; ^4^Genetics Program, North Carolina State University, Raleigh, North Carolina, USA

## Abstract

Iron deficiency (ID) and ID anemia (IDA) are global health concerns that tend to affect vulnerable populations, including women, children, and those living in areas disproportionately affected by environmental health hazards. A review of the literature was conducted using the top ten chemicals of public health concern as identified by the World Health Organization (WHO) in 2020, in combination with the terms “iron deficiency” and “anemia.” Both epidemiological and controlled experimental studies were considered. Eight contaminants or exposure classifications were ultimately considered to be within the scope of this review: lead, cadmium, arsenic, mercury, indoor and ambient air pollution, asbestos, dioxin and dioxin-like polychlorinated biphenyls (PCBs), and fluoride. Lead, cadmium, indoor and ambient air pollution, and fluoride are reliably linked to higher IDA prevalence and lower hematological parameters, including hemoglobin, hematocrit, and red blood cell count, all indicators of anemia. Direct measures of ID are less frequently reported. Further research studies, particularly controlled exposure studies, are needed to determine the importance of arsenic and mercury in contributing to the global ID and IDA burden. There is limited evidence that supplemental or dietary iron fortification can ameliorate the effects of lead, but not fluoride, and the efficacy of fortification has not been widely studied in the context of the remaining contaminants. Asbestos, dioxin, and dioxin-like PCBs are linked to anemia; however, the role of iron homeostasis is more complex and tends to include iron bioaccumulation. The narrative review has identified a need for renewed effort to address environmental factors beyond diet and nutrition when implementing ID and IDA interventions.

## 1. Introduction

In 2020, the World Health Organization (WHO) released a list of the top ten contaminants or contaminant groups of public health concern, including air pollution, arsenic, asbestos, benzene, cadmium, dioxin and dioxin-like substances, fluoride, lead, mercury, and pesticides [1]. Of these, eight have been linked to iron deficiency (ID) or anemia. Anemia is classified as one of the top WHO global health targets [2]. A quarter of the global population is anemic, and anemia is responsible for 660 years lived with disability per 100,000 people [3]. Providing additional nutritive iron is not always an effective treatment, so mitigating exposure to key chemicals will be necessary to tackle the global disease burden [3].

The scope of this review is a summary of the current understanding of the effects of exposure to key chemicals of global health concern on ID and ID anemia (IDA) (ID/IDA) using the last twenty years of research. Toxic metals (lead, cadmium, mercury, and arsenic), air pollution (indoor and ambient), asbestos, dioxin and dioxin-like compounds (DLCs), and fluoride will be discussed. Though there is robust evidence that benzene causes aplastic anemia, the condition is caused by damage to the bone marrow and is unrelated to iron homeostasis, so this topic is outside the scope of this review. Pesticides are similarly not implicated in iron-related anemias.

We developed the following literature search strategy, using PubMed and Google Scholar as our databases, to conduct a rigorous nonsystematic narrative review. The following search terms were entered using a strategy of pairing each exposure term (lead/Pb, cadmium/Cd, mercury/Hg, arsenic/As, air pollution, PM2.5, PM10, dioxin, 2,3,7,8-tetrachlorodibenzo-*p*-dioxin (TCDD), DLCs, polychlorinated biphenyls (PCBs), and fluoride) with the terms “anemia” and “iron deficiency.” Observational studies of human populations or wildlife were selected using the following inclusion criteria: a reported anemia diagnosis, systemic hemoglobin levels, systemic iron levels, systemic ferritin levels, and/or red blood cell counts, and documented exposure to the toxicant or toxicant group of interest, either by self-reported use, investigator-reported use, ecological probability, or quantification in biofluids. Experimental in vivo and in vitro studies were included based on the criteria that the study was designed to deliver a controlled quantity of the chemical of interest to an animal model or mammalian cell line and reported elemental iron, ferritin and/or hemoglobin quantitation, and/or expression of genes or proteins with key roles in iron absorption and transport. In order to provide a contemporary perspective, only articles published since 2003 have been considered. The aim of this review is to determine which of the global priority pollutants have sufficient evidence to be implicated in ID/IDA, identify gaps in understanding, and discuss a role for iron supplementation intervention.

## 2. Toxic Heavy Metals and Metalloids

The relationship between toxic metal and metalloid exposure and ID/IDA can be difficult to study in populations because of the complicated interplay between the two factors. ID is both induced by and increases the absorption of certain heavy metals, and disentangling the direction of influence is a challenge, especially from cross-sectional epidemiological studies. The perinatal period appears to engender special vulnerability to these phenomena because maternal and child health can be significantly impacted by the interaction between metal burden and ID.

### 2.1. Lead

#### 2.1.1. Observational Studies

Epidemiological studies have determined associations between blood lead levels and parameters associated with ID/IDA (Table 1). Interpretation of these findings differs among researchers, and it remains unclear if the primary directionality of the relationship is that lead exposure causes ID or ID increases lead absorption. Among pregnant women, higher blood lead levels have frequently been linked to ID/IDA with poor birth outcomes; however, one study of mothers found no association between lead in breast milk or infant hair with maternal anemia status [4–6, 29]. Higher blood lead levels in infants and children are associated with lower hemoglobin and blood iron levels and higher anemia prevalence. A cross-sectional study of infants found an association between lead levels and ID [7]. Exposure to lead from e-waste processing inhibited hemoglobin synthesis in children, and those living in a region with especially high lead exposure leading to blood lead levels over 100 μg/L have higher rates of anemia [8, 9]. In addition to those in lead hotspots, children living in cities with higher blood lead were more likely to have lower hemoglobin levels, suggesting urban lead exposure is also linked to iron status [10, 11]. This association may also exacerbate the effects of other illnesses, which has been found in children with malaria [12]. In contrast, several studies of otherwise healthy children found no association with iron status or anemia, and one found a positive correlation between blood lead and iron [13–17].

Although there is a sensible emphasis on studying the health impacts of lead on mothers and children, adult lead exposure, both in industrial and everyday settings, also contributes to disease development. Lead exposure or biomarkers of lead toxicity in factory workers have been linked to higher anemia prevalence among otherwise healthy men and women [18–20]. Like children, adults living in areas with a history of smelting activity are at risk for lead toxicity, including anemia [21]. Adult lead-associated anemia was also found among those who ingested ayurvedic medicines, unregulated herbal supplements traditionally used in India [30]. In contrast, one study of adults found no such association between blood lead and iron status [22]. Although interpretation of causality from cross-sectional studies is typically limited, the U.S. Environmental Protection Agency (EPA) attempted to determine the directionality of the relationship between lead exposure and anemia. Researchers compared the geolocation of measurable lead levels in drinking water with hemoglobin levels in patients with chronic kidney disease and found that patients with lead-contaminated drinking water had reduced hemoglobin [23]. Because this study methodology involved testing lead levels at the exposure source, researchers interpreted these findings as evidence that lead exposure may have caused the lower hemoglobin in this population. Interestingly, an environmental study of Nile crocodiles also found that animals with higher lead burden exhibited signs of anemia, suggesting the effect is not specific to mammalian hematology [31]. Environmental sampling has not always yielded an association between lead levels and blood parameters, including a study of wood mice in a polluted region with historical industrial activity [32].

#### 2.1.2. Experimental Studies

Controlled experimental studies of lead exposure have the potential to provide clarity on the precise nature of the relationship between lead and anemia (Table 2). After nearly a month of lead acetate administration, male rabbits and male rats both exhibited signs of anemia [33, 34]. Nine weeks of lead acetate exposure in rats led to decreased iron levels in blood and kidneys, but increased iron (and other micronutrient metals) in the urine, suggesting targeted metal excretion is partially responsible for these deficiencies [35]. Despite the prevalent focus on developmental outcomes of lead exposure in the public consciousness, few experimental studies have investigated the impact of early life exposure to lead on anemia, though one study did find that aging mice accumulated iron in brain tissue after developmental lead exposure, though systemic iron levels were not reported [36]. In addition to the evidence from environmental sampling of crocodiles mentioned above, one group looked at cockerel chicks and found that administration of lead acetate induces anemia, further supporting conservation of this association in nonmammalian species [37]. However, one study in mice found no impact of lead exposure on hemoglobin [38]. Providing iron supplementation to schoolchildren in Mexico with high blood lead levels did reduce lead burden over time but did not improve measures of cognitive ability [46]. These findings indicate preexisting ID worsened lead absorption in this population, and that iron supplementation can reverse lead accumulation but not the cognitive effects.

### 2.2. Cadmium

#### 2.2.1. Observational Studies

Cadmium burden typically correlates with markers of ID/IDA such as serum hemoglobin, total body iron, and serum ferritin in adults, including mothers in Turkey, Ohioan adults, participants in the 2003–2008 National Health and Nutrition Examination Survey (NHANES), and Iranian adults [5, 22, 24, 25] (Table 1). However, a few studies have found that blood cadmium did not correlate with ID/IDA although the researchers noted overall cadmium levels were low in one of the studied populations [16, 17, 21]. Samples collected from wood mice indigenous to an area with a history of smelting revealed higher cadmium burden was associated with lower hematocrit, suggesting higher anemia risk extends to wild mammalian populations [32]. In a rare example of longitudinal observation, preexisting ID was not found to stimulate female farmers to absorb more cadmium [26].

#### 2.2.2. Experimental Studies

The impact of cadmium exposure on various parameters related to ID/IDA has been widely studied in rodent models (Table 2). Short-term (3 weeks), high-dose (100–200 ppm cadmium chloride) exposure in drinking water did not induce iron imbalance in mice [43]. In contrast, long-term oral cadmium exposure consistently leads to deficient iron status. Exposure for up to nearly two years via high dose (300 ppm) dietary augmentation induced marked hepatic hypoferremia, which appeared to be caused by reduced intestinal iron uptake [39]. Renal hypoferremia also occurred after one to six months of exposure to 50–75 ppm cadmium chloride in drinking water and was linked to renal oxidative stress and chronic kidney injury [40]. Iron metabolism and import-related genes were dysregulated in the kidneys, including upregulation of *Lrp2, Slc39a14*, and *Slc39a8* after 1 month and downregulation of *Cubn, Lrp2, Slc39a14*, and *Slc39a8* at 6 months, suggesting duration of exposure impacts the nature of iron metabolism dysregulation [40]. Interestingly, coadministering apocynin, an antioxidant, improved anemia-related blood parameters, suggesting cadmium-induced IDA is related to oxidative stress [41]. Cadmium-induced ID also occurs with developmental exposure and impacts the developing offspring. Two months of maternal cadmium exposure through drinking water (1–50 ppm) before and during gestation led to maternal and newborn ID and hepatic hypoferremia [42]. Offspring exhibited reduced birthweight and cardiovascular abnormalities, two features associated with ID in humans [42, 47].

Although few in vitro studies examine the relationship between cadmium and iron, iron content was reduced in primary renal proximal tubule (rPT) cells treated with 1.25–10 μM cadmium [40]. In HeLa cells, cadmium exposure downregulated the expression of transferrin receptor genes and iron regulatory proteins, suggesting a lack of available machinery for iron transport and storage [43]. However, interpretation of in vitro studies is limited because cadmium bypasses intestinal or respiratory absorption in cultured cell platforms, and systemic ID/IDA cannot be evaluated.

Similar to the findings from lead exposure studies, iron and other micronutrient metals were more abundant in the urine of cadmium exposed rats, again suggesting that upregulation of metal excretion is partially responsible for decreased physiological iron availability seen with toxic metal exposure [35]. Additionally, long-term cadmium exposure led to iron deposition in the bones of rats which may contribute to iron depletion seen in other organs and blood [44].

### 2.3. Mercury and Arsenic

Compared to lead and cadmium, very few controlled experimental studies have been conducted to investigate the impacts of mercury or arsenic on ID/IDA (Table 2). Unlike cadmium, mercury levels in breast milk or infant hair were not implicated in maternal anemia status or hemoglobin levels [5] (Table 1). Globally, children and adults are at high risk of mercury exposure when they live near or work in the unregulated gold mining industry. In areas of the Peruvian Amazon where gold mining is an important income source, exposure to methylmercury via contaminated fish led to an inverse association between hair mercury and hemoglobin in children under twelve [27]. In this population, it is unclear if preexisting anemia rendered children more vulnerable to mercury absorption, or mercury exposure caused the low hemoglobin levels [27]. In support of the conclusion that methylmercury exposure can lead to anemia, Nile tilapia exposed to doses of methylmercury chloride ranging from 0.5 to 2 mg/kg-diet for 60 days exhibited lower hemoglobin levels, reduced red blood cell count, and upregulation of hepatic metallothionein, suggesting heavy metal toxicity and anemia [48]. Unlike lead, cadmium, and mercury, all of which are predominantly released into the environment through anthropogenic activities, arsenic is naturally found in soil and groundwater hotspots throughout the world in a manner that is difficult to remediate. In contrast to mercury, arsenic burden was not associated with anemia incidence in people or sheep [14, 28] (Table 1). However, rats given 100 ppm arsenic for 10 weeks developed low hemoglobin, a phenotype that was successfully rescued by the administration of chelators [45] (Table 2).

### 2.4. Summary

It is clear that body burden of heavy metals and metalloids is frequently associated with hematological biomarkers of ID and anemia in adults, children, and both wild and experimental animals. Exposure to cadmium, and to some extent lead, tends to both decrease intestinal iron uptake and encourage the transport of iron from blood, liver, and kidney compartments into the bones and urine, reducing its bioavailability. The hypothesis that ID/IDA itself increases the absorption of metals due to compensatory upregulation of divalent cation uptake was supported in the context of lead but remains unsupported in cadmium. Although mercury and arsenic have been linked to anemia at the population levels, there is insufficient experimental evidence to determine a causal relationship.

## 3. Particulate Matter (PM) and Other Nonmetal Air Pollutants

Poor air quality is one of the most common toxic exposures, with 99% of the global population living in areas where at least one pollutant exceeds WHO recommendations [49]. Key health concerns associated with air pollution include asthma, cardiovascular diseases, and cancer [50]. Despite evidence that air pollution is also linked to anemia, it is not currently emphasized by WHO as a significant risk [49]. While outdoor air quality has been vastly improved in the last few decades, the risks of indoor air pollution are often overlooked and unregulated despite most people spending the majority of their day inside [50].

### 3.1. Indoor Air Pollution

Globally, solid fuels like coal, wood, and animal dung provide energy for indoor cookstoves and are often used in small unventilated homes. Women and children are particularly vulnerable as the household members that traditionally spend more time indoors than men [51]. Based on survey responses, women who report using biomass fuels, defined as plant or animal waste material, for indoor cooking were at greater risk for anemia during pregnancy than those who used so-called clean fuels like electricity or gas [52–54] (Table 3). However, one survey conducted among nonpregnant women did not suggest any correlation between cooking fuel type and anemia or blood hemoglobin [55]. In addition to survey data, direct measurement of indoor air PM in the homes of women along with blood sampling found that household PM2.5 and PM10 levels negatively correlated with hemoglobin levels [51]. Commercially available air purifiers, a feasible household-level intervention, were effective at improving both household PM concentrations and hemoglobin levels in women [78]. Women in one rural community exhibited no association overall between the use of indoor cooking fires and hemoglobin, but surprisingly, among a subset of iron-deficient women, indoor fire use was linked to higher hemoglobin, suggesting that measuring hemoglobin alone is not sufficient to evaluate the health status of those exposed to indoor air pollution [56].

Children living in homes in rural areas where unclean cooking fuels, including kerosene, coal, charcoal, wood, and plant and animal matter, were predominantly used were more likely to have anemia, though there was a joint effect of fuel type with rurality [57, 58]. Exposure to smoke from biomass fuels at both the country level and the household level correlated with higher anemia prevalence [60, 61]. The use of biomass fuel tends to correlate with several potential factors with joint or confounding effects, including household socioeconomic status and rural or urban setting [57, 60, 61]. Another source of indoor PM and other contaminants is secondhand, or passive, tobacco smoke. Children who live in households where one or more adult resident smokes are also more likely to be anemic [62].

Radon gas is another type of indoor air pollutant, that, unlike indoor cookfires, is not anthropogenic. Released from the radioactive decay of underground uranium and thorium deposits, radon contamination of household air occurs in hotspots worldwide, including Mexico, Sweden, and Czechia. Found in households of all levels of socioeconomic status, even small quantities of radon gas can cause health effects like lung cancer, but the impact on anemia is not well studied [79, 80]. Beta particles, a highly penetrative type of radiation emitted by radon gas, in conjunction with PM and black carbon have been associated with decreased hemoglobin in elderly men [63]. The researchers suggest that because ionizing radiation can damage bone marrow, the process of erythropoiesis is impaired [63]. It is not yet known if radon exposure leads to ID/IDA.

### 3.2. Ambient Air Pollution

Regulation of ambient, or outdoor, air pollution is a top priority of global public health organizations. Epidemiological studies that compare regional levels of ambient air contaminants like PM, nitrogen dioxide (NO_2_), carbon monoxide (CO), and sulfur dioxide (SO_2_) to the individual prevalence of anemia, either through medical histories or direct measurement of biomarkers like hemoglobin and serum ferritin, have consistently found that people of all ages that live in more polluted air are more likely to be anemic and iron deficient [64] (Table 3).

In two studies of older adults, those exposed to PM2.5, NO_2_, ultrafine PM, engine combustion vapor, and black carbon were more likely to exhibit features of anemia, including lower hemoglobin and hematocrit [65, 66]. PM10, PM2.5, and PM1 levels in the community of residence were all associated with a greater risk of anemia diagnosis and with lower individual hemoglobin levels among adults [67]. PM2.5 was also associated with anemia among adults admitted to the hospital with another major illness [68]. Both mothers and children were more likely to have low hemoglobin levels if ambient PM2.5, PM10, SO_2_, or CO levels had been high during the third trimester of pregnancy [69–71].

Several studies conducted with young children also reveal a strong relationship between ambient PM and childhood anemia. Living in an area with high levels of ambient PM2.5 is a particularly strong risk factor for childhood anemia [72, 74, 75]. More in-depth analysis suggested the cumulative effect of exposure to constituent components of PM2.5 contributed to a 50% increased likelihood of anemia while the standard measurement of PM2.5 exposure, total PM2.5 mass, increased anemia odds by only 10%, suggesting that there is an additive effect of exposure to different types and sources of PM2.5 [76]. Secondary analysis of data from 36 countries confirmed this association is a global phenomenon [77].

### 3.3. Summary

Despite the robust and consistent evidence in humans, very few studies have been conducted to explain the causal factors that link both ambient and indoor air pollution with iron homeostasis and hematopoietic function. Suspensions of environmental PM2.5 samples were shown to induce hemolysis in vitro, and fulvic acid, a component of both tobacco and wood smoke, was shown to disturb iron trafficking in cultured respiratory epithelial cells, both of which result in ID and inflammation [81, 82]. In fact, several researchers postulate that chronic exposure to polluted air leads to sustained systemic inflammation [66, 74, 76]. Anemia of inflammation, also called anemia of chronic disease, occurs when physiologically available iron is low, despite adequate intake and storage, and is often linked to long-term illnesses like autoimmune disease or infection, but has not been examined in the context of environmental exposures [83].

## 4. Asbestos

Occupational asbestos exposure is commonly associated with pulmonary diseases and cancers, and there is developing interest in the role of iron in these pathologies [84] (Table 4). Workers with documented long-term asbestos inhalation exhibit elevated levels of extracellular iron and upregulated iron import machinery, and these finding are recapitulated in mice [85, 90]. Extracellular iron overload itself is linked to mesothelioma initiation [91, 92]. However, experimental evidence suggests asbestos-induced disruption of iron homeostasis is more complex because silicate fibers, the predominate component of industrial asbestos, bind and sequester iron with high affinity and extracellular iron accumulation may result in functional intracellular ID [86, 93] (Table 5). Exposing both rodents and primary human bronchial epithelial cells to silica particles results in rapid chelation of intracellular iron by the fibrous particles, along with the generation of reactive oxidants and activation of apoptosis pathways [94, 95]. Providing additional sources of available iron ameliorates the sequestration effect [94, 95].

### 4.1. Summary

The high affinity of asbestos for binding iron interferes with normal iron trafficking. Asbestos exposure leads to an imbalance between the iron levels of the extracellular and cellular spaces. While the extracellular iron overload is linked to asbestos-induced cancers, cellular ID impairs lipid metabolism, detoxification, and mitochondrial function [109]. Increasing the bioavailability of iron, however, can overcome this process [84].

## 5. Dioxin and Dioxin-Like Substances

TCDD (or “dioxin”) and DLCs, including 12 congeners of PCBs, are of particular concern because they do not break down and have become ubiquitous in the environment. Because of their environmental persistence, dioxin and DLCs have been detected in most life forms. Accumulation in humans and wildlife is linked to toxicity of the immune, reproductive, and hepatobiliary systems, and dioxin is a long-established iron chelator [110, 111]. Despite this knowledge, very few observational studies have been conducted to determine the association between dioxin or DLCs and ID and anemia. Two ecological studies found no association between hemoglobin and hematocrit levels in loggerhead sea turtles and their dioxin-like PCB burden, although the dioxin-like congeners were not universally detected in these populations [112, 113].

### 5.1. Experimental Studies

Anemia is a frequently reported consequence of dioxin exposure in rodents (Table 5). Rats treated orally with heptachlorodibenzo-*p*-dioxin (HpCDD) exhibited severe and sometimes lethal anemia, and mice treated with TCDD also displayed anemia and disrupted expression of genes related to heme metabolism and iron homeostasis [96, 114]. However, unlike what is observed in the context of heavy metal exposure, increasing available iron exacerbates the effects of dioxin, as iron accumulation and hepcidin repression have also been found [97, 115]. Dioxin-induced anemia is similarly documented in nonrodent models. Rainbow trout and goats fed TCDD-contaminated food developed anemia and reduced expression of the hemoglobin gene *HBB1*, and goats orally treated with TCDD also developed anemia [98, 99]. Dioxin-like PCBs, including PCB-77 and PCB-126, similarly induce anemia, but not ID. PCB-77 leads to anemia characterized by hepcidin suppression, increased serum iron, and reduced hemoglobin in mice and salmon larvae [100, 101]. PCD-126 induces a similar profile in mice and the American mink [102, 103].

### 5.2. Summary

Although dioxin and DLCs consistently lead to anemia in experimental settings, iron levels are generally increased in these models, not decreased. Dioxins and DLCs are also known to cause inflammation, and anemia of inflammation is often characterized by low levels of hemoglobin in the context of adequate iron stores [116]. Dioxins and DLCs do not generate ID/IDA, but these findings prompt further research into the potential link between dioxin-induced inflammation and inadequate hemoglobin levels.

## 6. Fluoride

Fluoridated water is considered one of the greatest public health advancements in the dental field and is effective at preventing tooth decay and dental caries [117, 118]. However, there are hotspots throughout the globe, particularly in Southern Asia, with high levels of naturally existing fluoride in ground water and mineral deposits. Excess fluoride, or fluorosis, is most commonly linked to bone malformations and dental staining, but development or exacerbation of anemia has also been documented in humans and experimental animals [119, 120]. Fluorosis can cause ID/IDA by reducing intestinal iron absorption but is also known to directly damage red blood cells [89].

### 6.1. Observational Studies

Those who live in areas where drinking water contains high levels of fluoride (> 1.0 mg/L) are at risk for developing fluorosis, which can include ID/IDA (Table 4). Adults in fluoride endemic regions display both ID, characterized by low corpuscular hemoglobin, and IDA, characterized by low hemoglobin and hematocrit [87]. Children only exhibited ID, suggesting they had not had fluorosis for long enough to progress into anemia [87]. Women in fluoride endemic regions are more likely to have IDA in pregnancy and give birth to low-birth-weight babies [88]. Diagnosed ID combined with fluorosis leads to lower hematological values than either condition alone [89]. However, since most studies in the field are not longitudinal, it is often unclear whether fluorosis or ID/IDA tends to precede the other. Iron supplementation is not sufficient to ameliorate fluorosis-induced ID/IDA. Despite dietary iron fortification, children who live in areas where popular food seasonings contain calcium fluorite maintain ID/IDA status unless these sources of fluoride are removed from their diets [121, 122].

### 6.2. Experimental Studies

A variety of animals exposed to fluoride in laboratory settings also develop ID/IDA (Table 5). Rabbits treated with 10–50 mg/kg sodium fluoride develop anemia characterized by reduced red blood cell count [104], and goats respond likewise to similar treatment regimens [105]; ID parameters were not measured in these studies. Broiler chicken exposed to very high doses of sodium fluoride (800–1200 mg/kg) also develop anemia [107]. However, conflicting findings have been reported in similarly exposed rats: one study reported reduced hemoglobin and red blood cell count, while the other reported no effect of fluoride treatment [106, 108].

### 6.3. Summary

While low-level water fluoridation and periodic fluoride treatment is important for sustaining dental health, excessive amounts have been consistently linked to the development of ID/IDA. Unfortunately, iron supplementation does not improve outcomes. Removal of environmental sources of fluoride, like drinking water and certain seasonings, is required to treat and prevent fluorosis-induced anemia.

## 7. Discussion and Conclusion

There is sufficient evidence that two of the priority toxic metals, lead and cadmium, can cause ID/IDA. Twelve cross-sectional studies of global populations detected an association between lead exposure and ID/IDA [4, 6–12, 19–21, 23]. Six cross-sectional studies found no association between lead exposure and ID/IDA [5, 13–16, 22]. Two studies conducted among adults and children living or working in proximity with industrial contamination sites found a positive correlation between circulating lead and iron, so these findings likely represent coabsorption of anthropogenically released metals [17, 18]. Experimental findings further strengthen this assertion. Five controlled experimental studies in mammalian and avian models found that one to three months of lead exposure produced reduced systemic iron and/or at least one feature of anemia [33–35, 37, 38]. Only one rodent study reported increased cerebral and circulating iron in aged offspring after prenatal lead exposure, which may represent developmental programming of iron homeostasis dysregulation [36]. Iron fortification has been shown to be effective in reducing lead burden but did not reverse the lead-induced cognitive delays in children [46]. There is an urgent need for more and earlier intervention programs that provide iron fortification to children in lead-endemic areas. Fewer cross-sectional studies have measured cadmium exposure and parameters related to ID/IDA than those that have examined lead exposure. In total, seven studies investigated this association cross-sectionally, five of which found evidence of increased odds of anemia diagnosis, or at least one biomarker of ID/IDA [7, 16, 22, 24, 25]. One longitudinal study found that preexisting ID did not change the way women absorbed cadmium from their occupational exposure as farmers [26]. These findings provide context to cross-sectional studies, suggesting that when ID and higher cadmium burden coincide, the causal mechanism is not that ID causes increased cadmium absorption. Cadmium and ID/IDA have been experimentally investigated more often than lead; ten in vivo and in vitro studies fit the inclusion criteria. In four rodent and two mammalian cell models, iron levels were reduced after cadmium exposure, or at least one biomarker of anemia was detected [39–43]. Only one rodent study found no effect [43]. A possible mechanism of both lead- and cadmium-induced ID/IDA arises from evidence that iron accumulates in the urine and bones of exposed animals, while it is depleted from the blood, liver, and kidneys [35, 44, 36]. Evidence for mercury- and arsenic-induced ID/IDA is limited. Only one epidemiological study noted a decline in hemoglobin with mercury exposure [27]. Several biomarkers of IDA were detected in rats exposed to arsenic [45]. Overall, more research is required to determine the relationship of both mercury and arsenic to ID/IDA. Established cohorts should be leveraged to quantify mercury and arsenic levels in existing samples to enable reporting of associations with anemia prevalence and hematological parameters and provide sufficient data to support the design of in vivo experimental studies that interrogate the underlying mechanisms.

Overall, there is also sufficient evidence that air pollution causes ID/IDA. In total, 26 observational studies that examined indoor or ambient air pollution fit the inclusion criteria, and 24 found a significant impact on ID/IDA. One found no association, and one found that hemoglobin was elevated [55, 56]. Indoor cookstove and solid fuel use accounts for ten of these studies, eight of which found evidence that women and children living in homes with indoor cookstoves are more likely to have ID/IDA [52–54, 57–61]. Ambient air pollution, typically measured at the ecological level, accounts for 15 of the included articles, all of which found an increased ID/IDA prevalence or at least one biomarker of ID/IDA related to at least component of ambient air pollution [65–73, 76, 77]. Overall, air pollution of any kind is the priority chemical with the most consistent epidemiological correlation to ID/IDA. Unfortunately, there is a dearth of experimental studies that interrogate the directionality or mechanisms behind this association, though there is some evidence that in vitro exposure to components of air pollution exposure induces hemolysis and inflammation and can perturb iron trafficking [81, 82].

Asbestos displays a well-defined mechanism of interfering with the physiological availability of iron. Asbestos fibers sequester iron into the extracellular space and cells become deficient. Four experimental studies examined the effects of asbestos in lung tissue or cells on iron trafficking, two of which specifically studied the intracellular space. Cellular iron was found to be depleted, which could be rescued by administration of additional bioavailable iron [94, 95]. Treatment with iron supplementation may prevent asbestos-induced ID/IDA; however, this was gleaned from cell culture models, and no intervention should be implemented without first considering the impact on asbestos-related cancer development and progression. Dioxin is similarly an iron chelator, reducing iron's bioavailability. Dioxin has been known for decades to induce anemia because of its role in iron sequestration. Since 2003, experimental studies have linked dioxins or DLCs to anemia and/or iron dysregulation. Dioxin does not cause ID and in fact tends to increase iron levels, making these chemicals poor candidates for remediation by iron supplementation [97, 101, 103]. It is not clear if this iron is bioavailable or bound to PCBs. There is evidence that fluoride does induce ID/IDA, but it is not able to be relieved by supplementation [121, 122]. Three additional epidemiological studies all found that living in areas with very high levels of fluoride in ground water is a strong risk factor for ID/IDA [87–89]. Of five experimental studies, four exhibited the same trend as the observational studies, further strengthening the conclusion that fluorosis symptoms may include ID/IDA [104–106].

In conclusion, there is very consistent evidence that air pollution exposure causes ID/IDA, but a surprising lack of reports of intervention programs targeted to regions with indoor or ambient air pollution exposure. There is also sufficient evidence to link excessive fluoride, lead, and cadmium to ID/IDA, though direct measurements of ID, like circulating iron or ferritin, are less frequently reported. There is evidentiary support for iron supplementation programs to reduce lead absorption, though only from a single study. Asbestos, dioxin, and DLCs have a more complex relationship with ID/IDA because of their affinity for iron sequestration. Despite this, increasing iron bioavailability has been shown to be effective in the context of asbestos, but not dioxins or DLCs, though only in in vitro studies. This review has ultimately identified the following imperatives: implementation of iron fortification programs to prevent childhood lead poisoning, experimental studies of the utility of iron supplementation in alleviating cadmium- and air pollution-related ID/IDA, experimental studies to confirm observational findings related to mercury and arsenic, and exposure mitigation interventions in fluorosis-endemic areas and regions with dioxin-like PCB contamination.

## Figures and Tables

**Table 1:** Observational studies of the association between lead, cadmium, mercury, and arsenic and ID/IDA in human populations.

Study details	Exposure assessment	Relevant findings	Reference
Cross-sectional; 396 pregnant women; Pakistan	Lead; Blood	Hemoglobin, hematocrit, red blood cell count, mean corpuscular volume, ferritin, serum iron ↓ Mean corpuscular hemoglobin, total iron binding capacity ↑ Hepcidin ↔	[4]

Cross-sectional; 74 pregnant women, 46 infants; Turkey	Lead, cadmium, mercury; Breast milk, infant hair	Anemia prevalence, postpartum hemoglobin, postpartum iron supplementation use ↔	[5]

Cohort; 99 anemic pregnant women, 41 nonanemic pregnant women; India	Lead; Blood	IDA prevalence ↑ Hemoglobin ↓	[6]

Cross-sectional; 210 infants; South Korea	Lead, cadmium, mercury, arsenic; Blood	Lead ID, IDA prevalence ↑ Cadmium ID prevalence ↑ IDA prevalence ↔ Mercury and arsenic ID, IDA prevalence ↔	[7]

Cohort; 222 children living in proximity to e-waste, 204 control children; China	Lead; Blood	In e-waste proximity population: Hemoglobin ↓ In control population: Hemoglobin ↔	[8]

Cross-sectional; 11,541 children with high blood lead; China	Lead; Blood	IDA prevalence ↑	[9]

Cross-sectional; 268 children; Lebanon	Lead; Blood	IDA, low transferrin saturation prevalence ↑	[10]

Cross-sectional; 222 children; Uruguay	Lead; Blood	Hemoglobin ↓	[11]

Cross-sectional; 198 children; Uganda	Lead; Blood	Hemoglobin ↓	[12]

Cross-sectional; 330 children; Peru	Lead; Blood	Anemia prevalence ↔	[13]

Cross-sectional; 60 children; Peru	Lead, arsenic; blood	Hemoglobin ↔	[14]

Cross-sectional; 90 children; Morocco	Lead; blood, mine proximity	ID, IDA prevalence ↔	[15]

Cross-sectional; 100 children; Uganda	Lead, cadmium; blood	Lead Hemoglobin, ferritin, hepcidin, transferrin receptor ↔ Cadmium Transferrin receptor ↓ Hemoglobin, ferritin, hepcidin ↔	[16]

Cross-sectional; 1110 children; China	Lead, cadmium; blood	Elemental iron ↑	[17]

Prospective cohort; 40 male lead workers, 10 male control workers; Pakistan	Lead; Blood	In lead workers: Anemia prevalence ↑ In control population: Anemia prevalence ↔	[18]

Cross-sectional; 533 male and 218 female adult lead workers; Taiwan	Lead; Blood	In men: Hematocrit, hemoglobin, red blood cell count, mean corpuscular volume, mean corpuscular hemoglobin ↓ In women: Hematocrit, hemoglobin, red blood cell count, mean corpuscular volume, mean corpuscular hemoglobin ↔	[19]

Cross-sectional; 270 male adult lead workers; Poland	Lead; Blood	Elemental iron ↑	[20]

Cross-sectional; 72 exposed men and 102 exposed women, 49 control men and 98 control women; China	Lead, cadmium; blood, urine	In men: Blood lead Anemia prevalence ↑ Urine lead, blood and urine cadmium Anemia prevalence ↔ In women: Anemia prevalence ↔	[21]

Cross-sectional; 186 exposed adults, 90 control adults; United States	Lead, cadmium; blood	Lead Ferritin ↔ Cadmium Ferritin ↓	[22]

Ecological; 597,968 kidney disease patients; United States	Lead; community water	Hemoglobin ↓	[23]

Cross-sectional; 599 women; United States	Cadmium; blood and urine	Body iron ↓ ID prevalence ↑	[24]

Cross-sectional; 64 men and 56 women; Iran	Cadmium; blood	Ferritin ↓	[25]

Case-control; 38 women farmers; Japan	Cadmium; food, urine, and feces,	With preexisting ID: Cadmium absorption rate ↔	[26]

Cross-sectional; 83 children; Peru	Mercury; hair	Hemoglobin ↓	[27]

Cross-sectional; 14 men and 14 women; Iran	Arsenic; hair	Hemoglobin ↔	[28]

*Note:* Arrows (↑, ↓, ↔) indicate directional change of outcomes in relation to increasing levels of the exposure of interest.

**Table 2:** Experimental studies of the association between lead, cadmium, mercury, and arsenic and ID/IDA biomarkers in animal models.

Exposure	Model	Relevant findings	Reference
Lead	Male Wistar rat	Hemoglobin, hematocrit, red blood cell count ↓ Mean corpuscular volume, mean corpuscular hemoglobin ↔	[33]

Lead	Male rabbits	Hemoglobin ↓	[34]

Lead, cadmium	Female Sprague-Dawley rats	Urinary iron ↑ Serum, renal iron ↓	[35]

Lead	Sprague-Dawley rats	Cerebral iron ↑, blood iron ↑↔ ^∗^time dependent	[36]

Lead	Cockerel chickens	Red blood cell count, hemoglobin ↓	[37]

Lead	C57BL/6J mice	Red blood cell count, hemoglobin, hematocrit, mean corpuscular hemoglobin ↔ Mean corpuscular volume ↓	[38]

Cadmium	C57BL/6J mice	Liver iron ↓ Serum iron ↔ Unsaturated iron-binding capacity ↑ Total iron binding capacity↑ ^∗^arrow denotes trend, effect is time dependent	[39]

Cadmium	Male Sprague-Dawley rats, rPT cells	Renal, rPT cell iron ↓ ^∗^Arrow denotes trend, effects are time and dose dependent Renal expression of iron import genes ↓	[40]

Cadmium	Male Sprague-Dawley rats	Red blood cell count, hemoglobin, hematocrit ↓ Mean corpuscular volume, mean corpuscular hemoglobin ↔	[41]

Cadmium	C57BL/6J x Cast/EiJ mice, female adults, offspring	Adult maternal and neonatal offspring: Blood and hepatic iron ↓ Adult offspring: Blood and hepatic iron ↔	[42]

Cadmium	Female C57BL/6 mice, HeLa cells	In vivo Intestinal, hepatic, renal, and splenic iron, iron regulatory protein, iron response element binding ↔ in vitro Iron regulatory protein, iron response element binding, transferrin receptor transcript ↓	[43]

Cadmium	Ovariectomized female Sprague-Dawley rats	Osseous iron ↑	[44]

Arsenic	Male Wistar rats	Red blood cell count, hematocrit, mean corpuscular hemoglobin ↓ Mean corpuscular volume ↔	[45]

*Note:* ^∗^Arrows (↑, ↓, ↔) indicate directional change of outcomes in response to the exposure of interest.

**Table 3:** Observational studies of the association between indoor and ambient air pollution and ID/IDA in human populations.

Study details	Exposure assessment	Relevant findings	Reference
Cross-sectional; 732 pregnant women; Ethiopia	Kerosene or charcoal cooking fuel use; Interview/questionnaire	Anemia prevalence ↑	[53]

Cross-sectional; 10,961 nonpregnant women; Ethiopia	Biomass cooking fuel use: Interview/questionnaire	Hemoglobin ↓ Mild anemia prevalence ↑ Moderate-severe anemia prevalence ↔	[54]

Cross-sectional; 12,782 pregnant women; India	Biomass cooking fuel use; Interview/questionnaire	Mild anemia prevalence ↑ Moderate-severe anemia prevalence ↑	[52]

Cross-sectional; 382 nonpregnant women; Sri Lanka	Biomass cooking fuel use; Interview/questionnaire, breath carbon monoxide monitor	Anemia prevalence ↔	[55]

Cohort; 100 women; South Korea	Indoor PM2.5, PM10; Gravimetric analysis, sensors	Hemoglobin, hematocrit, mean corpuscular volume, mean corpuscular hemoglobin ↓	[51]

Cross-sectional; 185 exposed nonpregnant women, 89 nonexposed nonpregnant women; Guatemala	Smoky biomass-fueled cook stove use; Interview/questionnaire	Hemoglobin ↑	[56]

Cross-sectional; 123,186 children; Sub-Saharan Africa	“Unclean” cooking fuel use (kerosene, coal/lignite, charcoal, wood, plants, animal dung); Interview/questionnaire	Anemia prevalence ↑ ^∗^Effect modified by household rurality	[57]

Cross-sectional; 29,768 children, India	Biomass cooking fuel use; Interview/questionnaire	Anemia prevalence ↑	[58]

Ecological; Children; 193 countries	Solid cooking fuel use; Country-level secondary data	Anemia prevalence ↑	[59]

Cross-sectional; 1150 children; Eswatini	Biomass cooking fuel use; Interview/questionnaire	Anemia prevalence ↑	[60]

Cross-sectional; 4829 men, 16,221 women; China	Gas and solid cooking fuel use; Interview/questionnaire	Hemoglobin, red blood cell count, hematocrit↓ ^∗^Effect modified by socioeconomic status	[61]

Cross-sectional; 740 children; Jordan	Household secondhand smoke: Interview/questionnaire	Anemia prevalence ↑	[62]

Cohort; 1704 elderly men; United States	Radon, black carbon, PM2.5: Field monitoring	Hemoglobin ↓	[63]

Health impact assessment; 5837 children, pregnant women, elderly adults; Serbia	Air quality index; Radial basis functional network, air quality index mapping	Anemia prevalence ↑	[64]

Case-control; 60 middle-aged and older adults with COPD, 60 controls; China	PM2.5, black carbon, ultrafine particles, accumulated mode particles; Field monitoring	PM2.5 Hemoglobin, red blood cell count, hematocrit, mean corpuscular hemoglobin, mean corpuscular volume ↔ Ultrafine particles Hemoglobin, mean corpuscular hemoglobin ↓ Red blood cell count, hematocrit, mean corpuscular volume ↔ Accumulated mode particles Hemoglobin ↓ Red blood cell count, hematocrit, mean corpuscular hemoglobin, mean corpuscular volume ↔ Black carbon red blood cell count, hematocrit, mean corpuscular hemoglobin, mean corpuscular volume ↔	[65]

Cross-sectional; 421 older adults; United States	PM2.5, NO_2_; Spatiotemporal modeling	Anemia prevalence ↑ Hemoglobin ↓	[66]

Cross-sectional; 10,611 older adults; China	PM10, PM2.5, PM1, NO_2_; Spatiotemporal modeling	Anemia prevalence ↑ Hemoglobin ↓	[67]

Ecological; 252 cities; China	PM2.5, ozone; Field monitoring	PM2.5 Anemia prevalence ↑ Ozone Anemia prevalence ↔	[68]

Cross-sectional; 259,627 infants and children; India	PM2.5, NO_2_, SO_2_, solid fuel use (wood, coal, dung cake, crop residue); Field and satellite monitoring, survey	Anemia prevalence ↑	[69]

Cross-sectional; 6824 newborn-mother pairs; China	PM2.5, PM10, SO_2_ CO; Field monitoring	Hemoglobin ↓	[70]

Retrospective cohort; 5323 primiparous and 2609 multiparous pregnant women; China	PM2.5, NH_4_^+^, NO_3_^−^, SO_4_^2−^, organic matter, mineral dust, black carbon; satellite and field monitoring	Primiparous: All Hemoglobin ↔ Mineral dust Anemia prevalence ↑ PM2.5, NH_4_^+^, NO_3_^−^, SO_4_^2−^, organic matter, black carbon Anemia prevalence ↔ Multiparous: Black carbon, NO_3_^−^, PM2.5, organic matter Hemoglobin ↓ NH_4_^+^, SO_4_^2−^, mineral dust Hemoglobin ↔ PM2.5, NH_4_^+^, NO_3_^−^, SO_4_^2−^, organic matter, mineral dust, black carbon anemia prevalence ↔	[71]

Cross-sectional; 117,511 children; Sub-Saharan Africa	PM2.5, SO_4_^2−^ SO_2,_ ozone, organic carbon, black carbon, CO; Satellite monitoring	PM2.5, ozone Anemia prevalence ↑ SO_4_^2−^ SO_2,_ organic carbon, black carbon, CO Anemia prevalence ↔	[72]

Ecological; 29 states, 7 union territories; India	Solid cooking fuel, PM2.5; Satellite monitoring, survey	Solid cooking fuel Anemia prevalence ↔ PM2.5 Anemia prevalence ↑	[73]

Ecological; 22 representative households from each of 640 administrative districts; India	PM2.5; Satellite monitoring	Anemia prevalence ↑ Hemoglobin ↓	[74]

Cross-sectional; 139,368 infants and children; Peru	PM2.5; Satellite and field monitoring	Moderate-severe anemia prevalence ↑ hemoglobin ↓	[75]

Cross-sectional; 177,072 children; India	PM2.5, NH_4_^+^, NO_3_^−^, elemental carbon; satellite monitoring	Anemia prevalence ↑	[76]

Cross-sectional; 154,443 children; 36 countries	PM2.5; Satellite monitoring	Anemia prevalence ↑ Hemoglobin ↓	[77]

*Note:* ^∗^Arrows (↑, ↓, ↔) indicate directional change of outcomes in relation to increasing levels of the exposure of interest.

**Table 4:** Observational studies of the association between asbestos or fluoride and ID/IDA in human populations.

Subject	Exposure assessment	Relevant findings	Reference
Cohort; 14 male exposed workers, 10 male and female controls; Unspecified location	Asbestos; Employment history	Bronchoalveolar lavage fluid iron ↑	[85]

Cross-sectional; 9 male and 1 female shipyard workers with documented asbestos-related disease; Italy	Asbestos; Employment history	Iron content in lungs near asbestos fibers ↑	[86]

Cohort; 93 exposed children and adults, 42 age- and sex-matched controls; India	Fluoride; drinking water, serum, urine	Adults Red blood cell count (males only), hemoglobin, hematocrit, mean corpuscular volume, mean corpuscular hemoglobin ↓ Children mean corpuscular volume, mean corpuscular hemoglobin ↓ Red blood cell count, hemoglobin, hematocrit ↔	[87]

Prospective cohort; 720 exposed pregnant women in high fluoride endemic region, 720 controls in lower fluoride endemic region; India	Fluoride; drinking water, urine	Anemia prevalence ↑	[88]

Prospective cohort; 96 exposed children and adolescents in fluoride endemic region, 385 controls in nonendemic regions; Thailand	Fluoride; drinking water, cooking water, urine, serum	Hemoglobin, hematocrit, mean corpuscular volume, mean corpuscular hemoglobin ↓ Red blood cell count ↔	[89]

*Note:* Arrows (↑, ↓, ↔) indicate directional change of outcomes in relation to increasing levels of the exposure of interest.

**Table 5:** Experimental studies of the association between dioxin, DLCs, asbestos, and fluoride and ID/IDA biomarkers in animal models.

Exposure	Model	Relevant findings	Reference
Asbestos	C57BL/6 mice	Lung iron, abundance of iron transport/absorption/binding proteins (DMT1, Dcytb, ferritin) ↑	[90]

Asbestos	Rat tissue in vitro	Asbestos-bound hemoglobin, hemolysis ↑	[93]

Asbestos	BEAS-2B cells	Mitochondrial iron ↓ ^∗^Reversed by ferric ammonium citrate administration	[94]

Asbestos	Primary human bronchial epithelial cells	Extracellular iron, DMT1 expression ↑ Intracellular iron ↓ ^∗^Reversed by ferric ammonium citrate administration	[95]

TCDD	C57BL/6J mice	Hemoglobin, red blood cell count, hematocrit ↓ Mean corpuscular volume ↔	[96]

TCDD, PCB126	C57BL/6 mice	TCDD Serum iron, urine iron, total iron binding capacity, hepatic transferrin receptor, hepatic SLC40A1, TFRC ↑ Liver iron, transferrin saturation, duodenal SLC40A1, SLC40A2, hepatic FTH1, FTL1 ↔ Hepatic *Hamp1, Hamp2,* HAMP1, ACO1, IREB2, HAO-1 ↓ PCB126 Hepatic *Hamp1*, *Hamp2* ↓	[97]

TCDD	Female Baladi goats	Red blood cell count ↓ Mean corpuscular hemoglobin ↔	[98]

TCDD	Rainbow trout	*HBA, HBB1* ↓	[99]

PCB77	Salmon larvae	Anemia incidence ↑	[100]

PCB77	Female Balb/c mice, HepG2 cells, L-02 cells	In vitro Hepcidin transcript ↓ In vivo acute exposure Hepcidin transcript ↓ Serum, spleen, liver iron ↔ In vivo continued exposure Hepcidin transcript ↓ Serum iron ↑ Spleen, liver iron ↔	[101]

PCB126	American mink	Red blood cell count, hemoglobin, hematocrit ↓	[102]

PCB126	Female C57BL/6 mice, HepG2 cells	In vitro Hepcidin transcript, ferritin protein ↓ Ferroportin ↑ In vivo Hepcidin transcript, liver and spleen iron ↓ Serum iron ↑	[103]

Fluoride	European rabbits	Red blood cell count, hematocrit, mean corpuscular volume, mean corpuscular hemoglobin ↓ Hemoglobin ↓↑ ^∗^effects are dose dependent	[104]

Fluoride	Goats	Hemoglobin, mean corpuscular hemoglobin ↓ Hematocrit, red blood cell count, mean corpuscular volume ↔ ^∗^Arrow denotes trend, effects are time dependent	[105]

Fluoride	Male Sprague-Dawley rats	Red blood cell count, hemoglobin ↓ Mean corpuscular volume, mean corpuscular hemoglobin ↔	[106]

Fluoride	Broiler chickens	Red blood cell count, hemoglobin, hematocrit ↓	[107]

Fluoride	Wistar-albino rats	Red blood cell count, hemoglobin, hematocrit, mean corpuscular volume, mean corpuscular hemoglobin ↔	[108]

*Note:* ^∗^Arrows (↑, ↓, ↔) indicate directional change of outcomes in response to the exposure of interest.

## Data Availability

Data sharing is not applicable to this article as no new data were created or analyzed in this study.
